# Long-Term Treatment Strategies of Pediatric Multiple Sclerosis, Including the use of Disease Modifying Therapies

**DOI:** 10.3390/children6060073

**Published:** 2019-05-31

**Authors:** Mary Rensel

**Affiliations:** The Mellen Center, Department of Neurology, Cleveland Clinic, Cleveland, OH 44195, USA; renselm@ccf.org

**Keywords:** pediatric, multiple sclerosis, disease modifying therapy, no evidence of disease activity

## Abstract

Multiple sclerosis (MS) presenting in the pediatric years can lead to landmark disability levels younger in life than adult onset MS and so therefore early and effective treatment remains paramount for long-term outcomes. The goals of MS therapeutics in adults have widened to address multiple mechanisms: anti-inflammatory, neuroprotective, and myelin repair, yet the optimal paradigm for MS therapies in the pediatric population is not known. Pediatric onset MS add complexities due to the ongoing development of the central nervous system and the immune system. Clinical trials have led to an increasing number of pharmaceutical therapies for adult onset MS (AOMS), one POMS randomized controlled trial is completed and other trials are ongoing, yet due to the low prevalence of POMS, the dynamic landscape and risk management of the MS disease modifying therapies (DMT) it remains more difficult to complete trials in POMS. There is consensus that controlled clinical trials leading to appropriate and safe therapies for POMS are important for a multitude of reasons that include unique pediatric pharmacokinetics, short and long-term safety, developmental issues, clinical benefits, and regulatory approval. This review will focus on new treatment goals, paradigm, strategies, monitoring, compliance, and products in the long-term treatment of POMS. The discussion will focus on these new concepts and the published data related to DMT use in POMS. This review provides significant insight into new concepts of treatment goals and current approaches to enhance the lives of the POMS patients now and in the future.

## 1. Introduction

Multiple sclerosis (MS) presenting in the pediatric years can lead to landmark disability levels younger in life than adult onset Multiple sclerosis (AOMS) [1]. Early and effective treatment remains paramount to pediatric onset MS’s long-term outcomes. AOMS patients have increasing pharmacologic treatment options over the past 20 years and now controlled clinical trials in pediatric onset Multiple Sclerosis (POMS) have been legislatively mandated, leading to ongoing trials in POMS and one completed randomized double blind trial [2]. Current treatment of POMS most typically follows AOMS recommendations and treatment strategies, with the limitation of not addressing developmental stages of the patient and the developing immune system [3]. Treatment strategies and goals have been defined in AOMS and proposed in POMS [4]. Newer MS treatment strategies are gaining notoriety as a means of enhancing long term outcomes in MS. There is consensus that controlled clinical trials leading to appropriate and safe therapies for POMS are important for a multitude of reasons, including unique pediatric pharmacokinetics, short and long-term safety, developmental issues, clinical benefits, and regulatory approval [5]. This review will focus on new treatment goals, strategies, and products in the long-term treatment of POMS. The discussion will focus on these new concepts and the published data related to disease modifying therapy (DMT) use in POMS. This review provides significant insight into the new concepts of treatment goals and a current approach to enhance the lives of the POMS patients now and in the future.

## 2. Comprehensive Approach to POMS Care

Confirmation of the diagnosis of POMS is followed by a comprehensive approach to address the needs of the patient and family, including long-term use of DMT, social and school support, cognitive assessment, lifestyle assessment and modification as needed, symptom management, and mental health assessment and treatment. Typically, a team-based approach is needed to address these lifelong concerns of the POMS patient [6]. A POMS treatment team may include pediatricians, pediatric neurologists, neuroimmunologists, urologists, ophthalmologists, social workers, nurses, physical therapists, occupational therapists, neuropsychologists, health psychologists, cognitive therapists, nutritionists, and speech therapists. An MS Care Unit, a highly specialized treatment team, has been proposed due to the complexity of POMS DMT use and symptom management [7]. Treatment goals of POMS are similar to AOMS, yet there are additional POMS concerns. These unique concerns of the POMS patient are long-term DMT efficacy and safety, neurodevelopmental stage, pediatric pharmacokinetics, and pharmacodynamics. Cognitive dysfunction needs to be assessed and addressed early in the POMS population due to the threat of disabling cognitive outcomes [8]. Identification of the distinct injury and repair mechanisms unique to the younger patients may help to direct treatment paradigms and development of blood and imaging biomarkers, but for now the treatment recommendations vary widely.

## 3. Treatment Goals

Current MS treatment goals include modifying disease outcomes and maximizing patient safety. No evidence of disease activity (NEDA) is an accepted AOMS treatment goal referring to the absence of disease activity clinically and radiographically. NEDA has been redefined over the years to include more detailed disease outcomes. NEDA 3 has more detailed components: annualized relapse rate (ARR), number of new/newly enlarging T2 lesions (neT2), and no evidence of disease activity [9]. NEDA-3 was used a post hoc outcome in the adult RCTs of fingolimod treated versus placebo/Interferon β treated patients assessing outcomes of 3 RCT trials showing the strongest benefit in the youngest adult patients, ages 18–20, 21–30, or over 30 (all *p* < 0.05) and again proving NEDA as a realistic goal post for MS treatment [9]. NEDA 3 has further evolved to include cortical lesions (NEDA 3 + CL) [10]. Cognitive deficits are a common disabling symptom in POMS. AOMS cognitive deficits are associated with the presence of CLs. Inclusion of the cortical lesions in NEDA 3 + CL may be important in POMS where cortical lesions have been confirmed and may help to define POMS treatment goals [11].

## 4. Treatment Paradigms

The process of choosing a DMT should include a risk benefit discussion to clarify treatment goals of the provider, patient, and family. Clarifying individual’s priorities and trade- offs that may be acceptable to the patient and their family will focus the collective goals and risk tolerance. These priorities may be concordant or discordant between patient and provider and may depend on the patient and families’ understanding of the disease, neurocognitive status, and adherence to past medications [12,13]. Studies have shown the patient’s determinants of DMT choice include many variables: long term risk of disability, dosing, monitoring, cost, and safety of medication [14]. General practice guidelines and recommendations exist for DMT use in POMS, but the best treatment strategy is not known and is fluid, as more DMTs are available for use [15,16,17].

DMTs can be divided by their efficacy, mechanism of action, mode of administration, monitoring needs, safety, or by the length or frequency of the treatment [16]. DMT use should commence when the diagnosis is confirmed. Studies show frequent delays in treatment initiation in POMS. Mean DMT initiation delay of 20 months was seen in one POMS database review, which likely can lead to worsening disability due to ongoing inflammation and subsequent damage while awaiting treatment initiation [17,18]. Historically, the first line therapies for POMS have been injectable therapies including interferon β and glatiramer acetate supported by the AOMS and observational studies in POMS [19,20]. Long-term injectable DMT for relapsing forms of MS was first approved in the 1990s, see Table 1. From 2012–2019, nine new oral and infusion DMTs were approved for AOMS, enhancing the choices and pattern of use of DMTs for this population, see Table 2 and Table 3. The lack of randomized controlled trials (RCT) of DMT in POMS originally limited DMT use in POMS. One RCT has been completed in POMS, see Table 4 and many are ongoing. Currently there are 17 FDA approved DMTs, (see Table 5) for relapsing forms for AOMS and only one FDA approved DMT for POMS, and one FDA approved for adult onset primary progressive MS. EMA has approved Interferons and glatiramer acetate for patients 12 years of age and older. Canada has approved Fingolimod for POMS.

The pattern of use of DMT in POMS has changed with the availability of higher efficacy DMT. These previous “first line” therapies have frequent side effects, are more difficult, and have lower efficacy as compared to our newer DMT for AOMS [20]. The sequence of DMT varies according to provider’s experience and goals, patient’s preference and goals, payor, patient location, and even variability within one country can affect DMT choice [7]. The recent review of the United States’ Network of Pediatric MS Center’s database illustrated the change in pattern of DMT usage in POMS. The 2018 review included over 1000 POMS patients and the newer higher efficacy DMTs were used as first line in 42% of the POMS patients, the newer DMT included dimethyl fumarate (*n* = 102), natalizumab (*n* = 101), rituximab (*n* = 57), fingolimod (*n* = 37), daclizumab (*n* = 5), and teriflunomide (*n* = 3) showing the shift toward higher efficacy medications as first line for POMS in the US [17]. The DMTs are approved for use in AOMS at varying levels internationally (see Table 5 [29]). There are locations in the European Union and various payors in the US that dictate DMT algorithms. The high efficacy therapy protocol has been evaluated in uncontrolled smaller series in POMS, such as a Natalizumab series in 20 POMS patients showing the use of first line high efficacy DMT for POMS [30].

The trial of Fingolimod versus interferon β-1a in POMS is the first double blind, active comparator trial completed in POMS showing superior efficacy of fingolimod over interferon β-1a in reducing relapses in children and adolescents ages 12 to <18. The median duration of the trial regimen was 1.6 years. This trial confirmed POMS relapse rates were 2–3 times higher than AOMS [31]. The incidence of serious adverse events was higher in the fingolimod group as compared to the interferon β-1a group, serious infections were reported with fingolimod (see Table 6). Typical influenza-like symptoms were seen in the interferon group. There were 7 seizures reported in the trial, 6 with fingolimod, and 1 on interferon. Seizures have been reported more commonly in pediatric central nervous system demyelination syndromes, but this risk needs further monitoring. There were no reports of skin carcinoma that were seen in the AOMS trials with Fingolimod. Rebound disease, MS activity after cessation of fingolimod, has been reported in AOMS, yet not in POMS to date [32]. The discontinuation rates were lower for fingolimod as compared to interferon β-1a, half of the interferon β-1a. Discontinuation was commonly related to disease activity. The discontinuation rates related to adverse events were overall low: (Fingolimod 4.7 vs. IFN 2.8%) [2]. The fingolimod versus interferon β-1a trial is the first completed RCT in POMS and provides needed data addressing unique features of treatment issues in POMS.

Infusion therapies for MS have a longer lasting immune effect compared to oral DMT medications with variable windows of immune changes, natalizumab’s effects may last 4–16 weeks [26]. Alemtuzumab and Natalizumab have shown similar effects on relapse rates, and yet disability progression was less in Natalizumab as compared to Alemtuzumab, no controlled data in POMS [33]. Alemtuzumab has had effective pediatric use in heart and kidney transplants with known risks including immunosuppression and autoimmunity [34,35], and is currently being investigated as escalation therapy for POMS (ClinicalTrials.gov Identifier: NCT03368664). Rituximab has been assessed in a class 1 phase II study in AOMS showing significant improvement in relapse rate and MRI lesion load [36]. A reduction in relapse rate was shown in adolescents with POMS administered Rituximab [37]. The risk of severe infections including PML justifies randomized evaluation of these infusion therapies in POMS to clarify the long-term risk of these products in this unique population.

The dosing of certain pharmaceutical products in pediatric patients must take into account the unique absorption, distribution, metabolism, and elimination (ADME) of this age group [38]. The level of hepatic metabolizing enzymes may be significantly different in various age ranges and can differ between sexes as well. The renal glomerular filtration rate reaches adult values at 1 year of age and is therefore less of a concern [38]. The pharmacokinetics of a particular agent and potential differences according to the age of the child or adolescent must be taken into account due to variability. Pharmacodynamic studies in POMS are recommended as efficacy may be influenced by the differences in efficacy and tolerability according to the level of the developing immune system [38].

## 5. Monitoring Treatment in POMS

Monitoring of MS disease activity and DMT safety will depend on the DMT choice, the individual patient’s disease activity, and comorbidities (see Table 1, Table 2 and Table 3 for DMT monitoring needs). Due to risk of disability from POMS and concern over short-term and long-term safety of DMT POMS patients need regular clinical monitoring [1]. See Table 1, Table 2 and Table 3 for safety monitoring depending on DMT choice. Medication switches due to poor tolerance or noncompliance in POMS has been reported at a 16% rate in the first year of treatment [17]. An International Pediatric MS Study Group consensus statement defines inadequate treatment response in POMS: if the patient has been fully compliant on treatment for 6 months and demonstrates (1) no reduction in relapse rate or new T2 or contrast enhancing lesions (as compared to pretreatment); or (2) 2 or more confirmed relapses (clinical or MRI) within a 12-month period [38].

## 6. Escalation of Therapy

The optimal sequence of DMT is not known; there is lack of clarity on treatment sequencing in AOMS and POMS. There are uncontrolled studies of DMT used in the context of POMS showing similar efficacy as in the AOMS population. The terminology of first line versus second line is being replaced with the concepts of escalation and induction, as well as individualized therapy [4]. A 2011 open label multicenter series of POMS patients showed 55.8% of the patients with disease stability who therefore did not need to escalate therapy, while the rest of the patients needed 2–4 further DMT in 2 years due to refractory disease. The rate of change was similar if the patients started with Interferon or glatiramer [17]. The pattern of “first line” DMT choice is changing over time due to observational and controlled studies suggesting higher efficacy of newer DMT as compared with lower efficacy early approved DMT in AOMS and POMS, see primary and secondary outcomes of RCT in various DMT, see Table 7. The unique features of POMS have not been studied in all DMT [19]. The concept of utilizing high efficacy treatment in POMS from the disease onset has been proposed, the long term outcomes of which are not yet known [39]; the concern for long term disability in this population validates this proposal [40].

Escalation therapy refers to starting with low efficacy product and increasing the efficacy of the DMT depending on the NEDA status and tolerability. Comparison of the DMT AOMS studies has inherent limitations; multiple comparisons have been reported including a value based comparison from the Institute for Clinical and Economic review, ICER, looking at the strength of the evidence, patient goals, and costs for each FDA approved DMT products [47]. A standardized ICER evaluation of all MS DMT concluded the newest FDA AOMS approved product, ocrelizumab dominating the other DMTs when cost was compared with supportive care for the AOMS patient, showing it as the best value in the US market or as cost effective as a first line treatment for RRMS [27]. There is no similar ICER evaluation for DMT use in POMS. In the AOMS DMT ICER analysis Alemtuzumab dominated as the second line DMT over the other defined second line products: natalizumab, fingolimod and ocrelizumab for RRMS, providing more quality due to high efficacy and lower cost, while the other DMTs were similar in terms of cost and health outcomes. Maintaining up-to-date education on the changing landscape of DMT and risk tolerance also likely plays a role in the rate and timing of escalation therapy in various providers [28]. There has been global concern over the cost and access to DMTs, especially financial barriers to the patients and their families.

## 7. Immune Reconstitution

Immune reconstitution (IR) therapy, a “resetting” of the immune system, intermittent or noncontiguous therapy, applies to various medications and procedures. There have been suggestions that these noncontiguous AOMS therapies may indeed be cost effective, as well as improve quality of life [48]. Autologous stem cell transplantation for severe cases of POMS has been an early example of this type of IR therapy. Concerns over IR treatment related mortality in early studies predominated, yet there was evidence of long-lasting (3 years), change in the T cell repertoire as well as clinical stability in AOMS patients following autologous stem cell transplantation [49,50]. A recent AOMS randomized trial of myeloablative stem cell therapies (SCT) in relapsing AOMS showed delayed time to disease progression when compared to other DMT [51]. Mesenchymal stem cells (MSC) are present in various tissues including bone marrow, adipose and umbilical cord blood, and their immune altering capabilities have been exploited in various disease states. MSC are reported to have minimal known risk, potential immune alteration, and CNS regeneration, yet the lack of controlled studies in POMS precludes their current use in this population [52]. Pediatric neurologic conditions treated with MSC include autism, cerebral palsy, and spinal muscular atrophy (SMA). MSC use in pediatric SMA was noted to be low risk yet not long-lasting, as there was recurrence of symptoms in SMA. The recurrence of symptoms in pediatric SMA may mean that MSC may indeed be a “maintenance” therapy after all [53].

## 8. Clinical Trials in POMS

Due to concern over the lack of RCT data addressing the unique features of POMS, there is now a demand from the Federal (FDA) and European (EMA) regulatory agencies for a Pediatric Investigation plan for approval of a new biologic agent [38,54] The IPMSSG addressed the challenges of completing a RCT in the POMS population with the need for controlled trials to determine the safety and efficacy of the DMT in POMS in the IPMSSG consensus statement of 2012 [38]. The unique ethical issues of this population have been clarified: immunological immaturity, risk of community acquired infections, neurodevelopmental factors as well as short and long-term toxicities [55]. There are ongoing POMS clinical trials with dimethyl fumarate, teriflunomide and the infusion alemtuzumab looking at efficacy and safety of these products in POMS. DMT pattern of use in AOMS in regards to escalation versus early high efficacy DMT is being examined in two ongoing AOMS trials: (DELIVER-MS (NCT03535298) and TREAT-MS (NCT03500328) [56] yet the optimal approach for POMS is not known. The long-term safety of DMT use in POMS does not extend beyond the data of the fingolimod RCT in POMS.

## 9. Compliance and Adherence

Compliance and adherence of DMT are important issues in POMS as it involves both the patient and level of caregiver support. The POMS patients’ compliance and adherence need to be assessed regularly as a rate of 1–47% nonadherence has been seen, can lead to poor outcomes, and may be difficult to dramatically improve with behavioral interviewing or electronic monitoring [57,58]. The patient’s perception and preferences of the DMT’s risk, side effects, and dosing likely affect compliance as well. Compliance can have a long-term effect on disability outcomes as seen in a series of MS patients on injectable medication with the highest compliance reporting lowest disability progression [15,59]. Compliance with DMT for 6 months is assumed in the IPMSSG statement on DMT efficacy [38].

## 10. Biomarkers in MS

Biomarker is an objective measure of a biologic process or the pharmacologic response to a therapeutic intervention. Biomarkers may play a role in the DMT choice, DMT risk management, and predicting treatment response. Biomarkers would therefore help to effectively and efficiently tailor the care POMS patient and guide the caregiver and family. There are proposed biomarkers for the diagnosis of MS, the DMT choice, and DMT maintenance of the various disease modifying products, yet the majority of the data is in the AOMS (see Table 8 below). Neutralizing antibodies to the interferons have been known to lessen efficacy and are used to guide therapy with the current recommendation to stop interferons due to high titers of neutralizing antibody [60]. John Cunningham visual index, or JC index is used to gauge the safety of using Natalizumab due to risk of Progressive multifocal encephalopathy or PML. JC status, prior use of immunosuppressive products, and length of use of Natalizumab increase the risk of PML. If Biomarkers range from blood to CSF tests and include neutralizing antibodies leading to less efficacy of the DMT to serum or CSF evidence of neurotrauma, as that seen with Neurofilament light chain (NFL), a marker of white matter axonal injury. Glial fibrillary acidic proteins at elevated levels can also be seen in AOMS and in the future may be used as a biomarker of disease activity. POMS study showed high levels of NFL in the CSF of pediatric cases with acquired CNS demyelination [61]. A low cost, simple test, such as a blood test, to predict and/or monitor the treatment response which could enhance the long-term outcomes of the patient due to enhanced active monitoring of the MS activity would be welcome as it could minimize patient discomfort due to tests, as well as minimize their cost.

There are possible biomarkers to guide therapy choice and to determine disease activity; neurofilament light chain (NFL) is one such biomarker. NFL and glial fibrillary acidic protein (GFAP) concentrations in the serum and CSF have correlated with overall disease activity, reflecting a recent relapse, disease progression, or MRI activity with a new or active lesion and may reflect axonal injury [62,63]. A blood test as a means of MS disease assessment would be a novel and effective way of managing a chronic disease that now relies on expensive MRIs for assessment of subclinical disease activity.

## 11. Neuroregeneration in POMS

Demyelination, axon loss, and marginal tissue repair lead to eventual disability in MS. Acute axonal damage is more extensive in POMS as compared with AOMS [64]. New treatment targets include remyelination, neuroprotection, and/or neuroregeneration in MS. Pediatric MS can lead to disability and may take more years as compared with AMS, but it does cause early neurocognitive deficits and eventual physical disability as well. Improved neural repair has been postulated in POMS, but the mechanism is unknown. Remyelination is impaired in MS and intensified due to disease duration and advanced age. Remyelination has been tested in AOMS and a successful phase II remyelination trial with clemastine has been reported to possibly improve myelin and therefore nerve function in the optic nerve [65]. In AOMS, blood tests have revealed a product, Coco, a bone morphogenetic proteins antagonist. Related to remyelination failure, alternation of this antagonist may induce further repair [66]. There are vaccines in an early phase of trial aiming to use immune tolerance for neuroprotection. More research is needed to repair and prevent disability in POMS.

## 12. Conclusions

POMS patients have a growing armamentarium of therapeutic products to enhance their long-term outcome and improve their quality of life. POMS has unique features when compared with AOMS: more inflammatory disease, more frequent relapses, neurocognitive disability levels, rates of cerebral atrophy, quantity of axonal damage, and MRI lesion accrual [4]; recommended POMS therapies should address these unique features. Due to the complexity of POMS, it is best to seek out an MS Care unit with a team-based approach and knowledge of risk management with the multiple DMTs. Shared decision making, a discussion of the goals of the DMT and the goals of the patient and family are paramount for achieving long-term success with the treatment course. The treatment goals should be expressed early and defined regularly to enhance compliance. Risk of the DMTs can be severe, and priorities need to be defined; the DMTs with black box warnings need to be reviewed in detail with the patient and their family. Higher efficacy DMTs are now used more often in POMS, and as such, less disability and relapses are being reported. Complementary and lifestyle “treatments” and/or modification of higher risk environmental factors may also enhance long-term outcomes in POMS and can be considered in collaboration with needed pharmaceutical products [61]. There is one completed randomized controlled clinical trial in POMS confirming the efficacy and safety of fingolimod in this population. Biomarkers before and during treatments can help predict and monitor treatment response. There are ongoing clinical trials in POMS to add to the efficacy and safety data of more DMTs for this unique population. Newer treatment goals of nerve repair and/or remyelination have not been systematically studied in POMS, but are goals to work toward in order to lessen and/or repair disability. For now, identifying the patient and family goals, defining MS Care Unit’s treatment goals, optimal DMT management, and a healthy lifestyle can serve the patient and family in the long run.

## Figures and Tables

**Table 1:** Summary of injectable disease modifying therapy for pediatric-onset multiple sclerosis (POMS).

**Interferon Β 1a**	
Mechanism of Action	Shifts cytokine balance to a more anti-inflammatory profile; reduces trafficking of inflammatory cells across the blood brain barrier
Dosing	Dose titration to start 22 or 44 µg sq three times weekly; 30 µg im weekly; or 125 µg sq every other week
Side effect	injection-site erythema, influenza-like illness, pyrexia, headache, myalgia, chills, injection-site pain, injection-site pruritus, arthralgia and asthenia
Monitoring	CBC and LFT at baseline and q 6 months
	Pregnancy test prior to use, it is not recommended to get pregnant or nurse while on this product
Adverse events	depression
	elevated transaminases and thyroid abnormalities
	Neutralizing antibodies to interferons
POMS publications	[21]
POMS RCT	Ongoing, ClinicalTrials.gov Identifier: NCT03870763
**Interferon β-1b**	
Mechanism of Action	Shifts cytokine balance to a more anti-inflammatory profile; reduces trafficking of inflammatory cells across the blood brain barrier
Dosing	0.25mg sq every other day
Side effects	See Interferon β-1a
Monitoring	
Adverse events	
POMS publications	[22]
POMS RCT	none
**Glatiramer Acetate**	
Mechanism of action	Modulates function of antigen-presenting cells; induces differentiation of CD4 + T cells into Th2 cells
Dosing	20 mg sq every day or 40 mg sq three times a week
Side effects	injection-site reactions, chest pain, rash, dyspnea, and vasodilation
Monitoring	No blood work required
Adverse events	Lipoatrophy, skin necrosis
POMS publications	[23]
POMS RCT	none
**Daclizumab**	
Mechanism of action	Humanized monoclonal antibody to alpha subunit of IL-2 receptor, reducing IL–2-mediated activation of lymphocytes
Dosing	150 mg sq once monthly
Side effects	influenza, bronchitis, eczema, lymphadenopathy, nasopharyngitis, upper respiratory tract infection, rash, and dermatitis. Rash (7% to 11%), depression (7% to 10%), upper respiratory tract infection (9% to 17%), pharyngitis (25%), and elevated ALT levels (5% to 6%) http://www.accessdata.fda.gov/drugsatfda_docs/label/2016/761029s000lbl.pdf
Monitoring	Assess for Hepatitis B and C, Daclizumab is contraindicated in Hepatitis B and C
	Assess for LFT at baseline, preexisting hepatic disease or impairment (ALT or aspartate transaminase ≥2 times the upper limit) or history of autoimmune hepatitis is contraindicated
	Pregnancy test prior to use, it is not recommended to get pregnant or nurse while on this product
Adverse events	Autoimmune Encephalitis (removed from US market 2018)
	Colitis
	Elevated Liver enzymes
POMS publication	[24]
POMS RCT	none

Abbreviations: sq = subcutaneous, im = intramuscular, CBC = complete blood count, LFT = liver function test.

**Table 2:** Summary of oral disease modifying therapy for POMS.

**Fingolimod**	
Mechanism of Action	Sphingosine 1 phosphate receptor modulator
Dosing	0.25 or 0.5 mg by mouth once a day
Side effects	Headache
	Back pain
	Diarrhea cough
AE	Low ALC
	QTc prolongation
	Respiratory Infection
	High ALT
	Rebound disease activity
	Rare PML
	Bradycardia
	First- and second-degree block
	Rebound disease sinusitis back pain first-dose monitoring requirement, contraindications in heart disease, risk of macular edema skin cancers
Monitoring	contraindications in heart disease
	VZV serum titer, pre FDO
	OCT pre and 3 months post FDO use with caution with other medications that prolong QTc interval
	Requires FDO < 6 h of observation with first dose with hourly vitals
	EKG before and after FDO
	CBC with diff baseline, 3 months then every 6 months
	JC index
	Avoid live vaccines while on Fingolimod and for 2 months after it is stopped.
	Pregnancy test prior to use, it is not recommended to get pregnant or nurse while on this product
POMS publications	[9]
POMS RCT	Completed [2]
FDA approved in POMS	Approved in POMS 2018
**Dimethyl Fumarate**	
Mechanism of Action	Increase Nuclear factor erythroid 2 (NF-E2)-related factor 2
Dosing	120 mg oral twice a day × 7 days then 240 mg oral twice a day
Side effects	Flushing, diarrhea, nausea, vomiting, and abdominal pain
Adverse events	Low absolute lymphocyte count
	Rare PML case
Monitoring	Baseline CBC with diff and LFT then q 12 months
	Pregnancy test prior to use, it is not recommended to get pregnant or nurse while on this product
POMS publications	[25]
POMS RCT	Yes, ongoing BG00012, ClinicalTrials.gov Identifier: NCT02283853
	ClinicalTrials.gov Identifier: NCT03870763
FDA approved for POMS	No
**Teriflunomide**	
Mechanism of Action	Inhibits pyrimidine biosynthesis
Dosing	7 mg or 14 mg oral once a day
Side effects	Diarrhea
	Nausea
	Headache
Adverse events	Decreased hair density
	Elevated Alanine aminotransferase
	Increased blood pressure
	Paresthesia in the upper extremities
	Major birth defects and embyrolethality in animal studies relatively long half-life of approximately 19 days, elimination can take >8 months
Monitoring	CBC with diff, LFT baseline, LFT q month × 6, Blood pressure monitoring
	Effective birth control compliance in male and female patients
	Contraindicated in severe hepatic impairment and those of child bearing potential not using effective birth control (http://products.sanofi.us/aubagio/aubagio.pdf)
	Pregnancy test prior to use, it is not recommended to get pregnant or nurse while on this product
Rapid elimination due to long half life	If drug-induced liver injury or pregnancy is suspected, discontinue AUBAGIO and start an accelerated elimination procedure with cholestyramine or activated charcoal
POMS Publications	None
POMS RCT	Yes, ongoing ClinicalTrials.gov Identifier: NCT02201108
FDA approved for POMS	None

Abbreviations: ALC = absolute lymphocyte count, FDO = First dose observation, LFT = liver function tests, VZV = *Varicella Zoster* virus, PML = progressive multifocal leukoencephalopathy.

**Table 3:** Summary of infusion disease modifying therapy for POMS.

**Natalizumab**	
Mechanism of Action	Inhibits the transmigration of immune cells across the blood-brain barrier thus inhibiting inflammation in the central nervous system.
Dosing	300 mg Intravenous every 4 weeks or 3–5 mg/kg IV every 4 weeks
Side effects	Infusion reactions
	Headaches
	Fatigue
Adverse events	PML
	Infections
	Neutralizing antibodies
	JC antibodies
	Rebound disease
	Encephalitis
	Meningitis
	Acute retinal necrosis due to herpes infection
Monitoring	Risk evaluation mitigation strategy due to risk of PML
	Monitor for the risk of PML while on Natalizumab and at least 6 months after stopping Natalizumab
	Monitor patients for signs or symptoms of PML, if any concerning signs, hold treatment and evaluate the patient as needed, clinically, MRI and possibly cerebrospinal fluid for JC virus DNA
	John Cunningham virus index q 3 months
	CBC and LFT q 6 months
	Natalizumab antibodies
	Monitor 1 h after infusion
	Pregnancy test prior to use, it is not recommended to get pregnant or nurse while on this product
PML	Progressive Multifocal Leukoencephalopathy risk factors: Prior use of immunosuppressants Presence of anti-JCV antibodies Length of time on Natalizumab
POMS publications	[26,27,28]
POMS RCT	none
FDA approval in POMS	none
**Alemtuzumab**	
Mechanism of action	monoclonal antibody against CD52 and leads to depletion of mature lymphocytes
Dosing	12 mg IV daily × 5 year 1 then 12 mg IV daily × 3 in year 2 and then as needed depending on disease activity
Side effects	Infusion reactions rash, headache, vomiting, nausea, diarrhea, flushing, abdominal pain, pyrexia, nasopharyngitis, urinary tract infection, fatigue, insomnia, upper respiratory tract infection, herpes viral infection, urticaria, pruritus, thyroid gland disorders, fungal infections, arthralgia, extremity pain, back pain, sinusitis, oropharyngeal pain, paresthesia, and dizziness
Adverse events	Autoimmune conditions
	Infections
	Possible cancer
	Premedication needed for infusion
	Antiviral needed for risk of viral infection until CD4 > 200 cells/u/L
Monitoring	*Varicella Zoster* virus titers before infusion, vaccinate as needed
	All necessary immunizations, including varicella zoster vaccine, should be completed at a minimum of 6 weeks prior to administration of alemtuzumab
	risk evaluation mitigation strategy program due to risk of serious adverse reactions
	Continuous observation × 2 h after infusion
	Labs and urine month × 5 years after last infusion
	CD4
	Risk management strategy
	Pregnancy test prior to use, it is not recommended to get pregnant or nurse while on this product
POMS Publications	none
POMS RCT	Yes, ongoing (ClinicalTrials.gov Identifier: NCT03368664)
FDA approval in POMS	none
**Ocrelizumab**	
Mechanism of Action	Humanized form of the CD20 monoclonal antibody
Dosing	300 mg IV repeated in 2 weeks then 600 mg IV every 6 months.
	Pretreatment with IV steroids and Benadryl
Side effects	Infusion reaction
Adverse events	Upper respiratory tract infections (40% to 49%), lower respiratory tract infections (8% to 10%), infusion reactions (34% to 40%), and infection of the skin and/or subcutaneous tissue (14%) https://www.gene.com/download/pdf/ocrevus_prescribing.pdf [Reflist]
Monitoring	Risk evaluation mitigation strategy due to risk of autoimmune disorder risk and cancer risks
	Screen for Tuberculosis and Hepatitis B prior to use
	Vaccinate at least 6 weeks prior to therapy
	Monitor 1 h after infusion
	Infection
	Possible breast cancer
	Pregnancy test prior to use, it is not recommended to get pregnant or nurse while on this product
POMS Publications	None
POMS RCT	None
FDA approval in POMS	None

Abbreviations: IV = intravenous, RCT = randomized controlled trials, CBC = complete blood count, LFT = liver function tests, PML = progressive multifocal leukoencephalopathy.

**Table 4:** Randomized controlled trial of fingolimod versus interferon in POMS.

POMS outcomes Age 10–17 [2]	Interferon β-1a	Fingolimod
*n*	108	107
Dosing	interferon β-1a at a dose of 30 μg per week	0.5 mg per day (0.25 mg per day for patients with a body weight of ≤40 kg
Adverse events	95.3% serious AE *n* = 7 (infection, supraventricular tachycardia)	88%serious AE *n* = 18, 16.8% (infection, leukopenia)
	Convulsions *n* = 1, 0.9%	Convulsions *n* = 6, (5.6%)
	AE > 10%	AE > 10%
	Nervous system disorder 42%	Leukopenia
		Eye disorder
	Psychiatric 10.3	Gastrointestinal disorders 34%
	Headache 29%	Infection and infestation 59%
		Influenza 11%
		Respiratory tract upper 15.9%
		Nervous system disorder 43%
		Headache 31.8%
		Psychiatric 18.7%
	Skin disorders 16.8%	Skin disorders 18.7%
Completed trial 87.4%	88 (81.5%)	100 [93.5%]
Primary End Point, Adjusted Annualized Relapse Rate	0.67	0.12, 82% decrease compared with IFN B-1a, *P* < 0.0001
MRI		New T2 lesion, 53% absolute difference
Secondary End Point Annualized Rate of New or Newly Enlarging T2 Lesions	9.27	4.39 relative difference, 53%; *P* < 0.001
Reasons for Permanent Discontinuation of Medication	N = 11, lack of treatment effect	Elevated liver aminotransferase levels, macular edema, cardiac arrhythmias or electrocardiographic (ECG) abnormalities, and pregnancy

**Table 5:** Global disease modifying therapy dates of approval.

DMT	FDA	EMA	Canada	Australia
Interferons AOMS	1995, 2002, 2009, 2012, 2013, 2014 (pegylated)	1995	1995	1996
		1997		
		1998		
		2008		
		2014		
Interferons POMS		Age 12–adult		
Glatiramer Acetate AOMS	1996, 2014	2002	1997	1997
		2015		
Glatiramer Acetate POMS		Age 12–adult		
Fingolimod AOMS	2010	2010	2011	2011
Fingolimod POMS	2018 Age 10–17		2018 Age 10–17	
Siponimod AOMS	2019			
Siponimod POMS	none			
Daclizumab AOMS	2016, removed from market 2018	2016	xx	xx
Teriflunomide AOMS	2012	2013	2013	2016
Teriflunomide POMS	none			
Dimethyl fumarate AOMS	2013	2014	2013	2017
Dimethyl fumarate POMS	none			
Cladribine AOMS	2019	2017	2017	2010, removed from market 2011
				Reapproved 2019
Cladribine POMS	none			
Natalizumab AOMS	2004, returned to market in 2006	2006, 2011 SC	2006	2006
Natalizumab POMS	none			
Alemtuzumab AOMS	2014	2013	2013	2015
	reserved for those with inadequate			
	responses to 2 or more MS medications			
Alemtuzumab POMS	none			
Ocrelizumab AOMS	2017	2018	2017	2017
	RRMS	RRMS		
	PPMS	PPMS		
Ocrelizumab POMS	none			

Abbreviations: SC—second line, RRMS—relapsing remitting MS, PPMS—primary progressive MS, AOMS—adult onset Multiple Sclerosis, POMS—pediatric onset multiple sclerosis.

**Table 6:** FDA Black Box warning and contraindications.

**Natalizumab**	Progressive Multifocal Encephalopathy (PML)
	Contraindicated if history of PML.
**Teriflunomide**	Contraindicated in patients with pre-existing acute or chronic liver disease
	contraindicated in women of childbearing potential
	Hepatotoxicity and risk of teratogenicity
**Alemtuzumab**	Risks of serious and sometimes fatal autoimmune conditions, infusion reactions, and various malignancies.
	It is contraindicated in human immunodeficiency virus due to CD4 + lymphocyte count reductions
**Daclizumab**	Daclizumab is contraindicated in Hepatitis B and C or liver impairment
	Removed from US market due to Autoimmune encephalitis
	Risk of severe liver injury and immune-mediated disorders, such as skin reactions, lymphadenopathy, and noninfectious colitis
**Fingolimod**	Fingolimod is contraindicated: if there is a cardiac event or vascular event like a stroke or TIA, irregular heart rate in the past 6 months and or medication that slows the QTc
**Siponimod**	Patients with a CYP2C9*3/*3 genotype (4)
	In the last 6 months, experienced myocardial infarction, unstable angina, stroke, TIA, decompensated heart failure requiring hospitalization, or Class III/IV heart failure (4)
	Presence of Mobitz type II second-degree, third-degree AV block, or sick sinus syndrome, unless patient has a functioning pacemaker (4)

**Table 7:** Primary outcome in Randomized Controlled Trials in AOMS.

	Reduction in ARR	Relative Risk Reduction to Placebo	Reduction in Sustained Disability	Reduction in MRI New T2 Lesions	Reduction in New Gad Enhancing Lesions
Alemtuzumab		Not tested vs placebo			
Dimethyl fumarate [41] vs. placebo	53,48%	44%	34%, 38%	85%	90%
Fingolimod [42]	See Table 6 for POMS data	23%			
Natalizumab	68%		42%	83%	
Daclizumab (removed from the market willingly by Biogen)	45% compared with IFN β 1a	45–54%		54% new or new enlarging T2	
Ocrelizumab [43,44]	44, 46% compared with IFN β 1a	46–47%	Slow disability in PPMS		
Teriflunomide [45]		31%	29%	69% against placebo	Free from gad lesions 89% compared with placebo
Cladribine [46]	66%		Slowed 3-month disability 33%		86%

**Table 8:** Potential serum biomarkers in MS.

Product	Potential Biomarker
Interferon	Neutralizing antibody
Natalizumab	Natalizumab antibody
	JC index
	L Selectin
	Neurofilament light chain
Fingolimod	VZV status
Alemtuzumab	interleukin (IL)-21
	CD4
	CD19
DMF	ALC
All	Neurofilament light chain
	Chitinase3-like 1
	Glial fibrillary acidic proteins
